# Similar hypothyroid and sepsis circulating mRNA expression could be useful as a biomarker in nonthyroidal illness syndrome: a pilot study

**DOI:** 10.20945/2359-3997000000625

**Published:** 2023-05-29

**Authors:** Robson José de Almeida, Andréa Harumy de Lima Hirata, Luiz Antônio de Jesus Rocha, Miriam Duarte de Arruda Motta, Patricia Varela, Leonardo Martins, João Bosco Pesquero, Cléber P. Camacho

**Affiliations:** 1 Universidade Nove de Julho Programa de Pós-graduação em Medicina Laboratório de Inovação Molecular e Biotecnologia São Paulo SP Brasil Laboratório de Inovação Molecular e Biotecnologia, Programa de Pós-graduação em Medicina, Universidade Nove de Julho (Uninove), São Paulo, SP, Brasil; 2 Universidade Federal de São Paulo Escola Paulista de Medicina Departamento de Medicina São Paulo SP Brasil Centro e Laboratório de Doenças da Tireoide de Endocrinologia Molecular e Translacional, Divisão de Endocrinologia, Departamento de Medicina, Escola Paulista de Medicina, Universidade Federal de São Paulo, São Paulo, SP, Brasil; 3 Johns Hopkins University School of Medicine McKusick-Nathans Institute of Genetic Medicine Baltimore Maryland USA McKusick-Nathans Institute of Genetic Medicine – Johns Hopkins University School of Medicine, Baltimore, MD; 4 Universidade Federal de São Paulo Departamento de Biofísica Centro de Pesquisa e Diagnóstico Molecular de Doenças Genéticas São Paulo SP Brasil Centro de Pesquisa e Diagnóstico Molecular de Doenças Genéticas, Departamento de Biofísica, Universidade Federal de São Paulo, São Paulo, SP, Brasil

**Keywords:** Transcriptome, RNA, sepsis, thyroid, nonthyroidal illness syndrome

## Abstract

**Objective:**

Based on hypothetical hypothyroidism and nonthyroidal illness syndrome (NTIS) gene expression similarities, we decided to compare the patterns of expression of both as models of NTIS. The concordant profile between them may enlighten new biomarkers for NTIS challenging scenarios.

**Materials and methods:**

We used Ion Proton System next-generation sequencing to build the hypothyroidism transcriptome. We selected two databanks in GEO2 platform datasets to find the differentially expressed genes (DEGs) in adults and children with sepsis. The ROC curve was constructed to calculate the area under the curve (AUC). The AUC, chi-square, sensitivity, specificity, accuracy, kappa and likelihood were calculated. We performed Cox regression and Kaplan-Meier analyses for the survival analysis.

**Results:**

Concerning hypothyroidism DEGs, 70.42% were shared with sepsis survivors and 61.94% with sepsis nonsurvivors. Some of them were mitochondrial gene types (mitGenes), and 95 and 88 were related to sepsis survivors and nonsurvivors, respectively. *BLOC1S1*, *ROMO1*, *SLIRP* and *TIMM8B* mitGenes showed the capability to distinguish sepsis survivors and nonsurvivors.

**Conclusion:**

We matched our hypothyroidism DEGs with those in adults and children with sepsis. Additionally, we observed different patterns of hypothyroid-related genes among sepsis survivors and nonsurvivors. Finally, we demonstrated that *ROMO1*, *SLIRP* and *TIMM8B* could be predictive biomarkers in children's sepsis.

## INTRODUCTION

The nonthyroidal illness syndrome (NTIS) occurs when an extrathyroidal disease affects thyroid hormone concentration without the appropriate hypothalamic-pituitary-thyroid (HPT) axis response (1). NTIS is the leading cause of thyroid hormone metabolism disturbed in hospitalized patients and could be a critical step in increasing their survival. A critically ill patient's thyroid function is affected by diseases (thyroid-originated or not) and drugs (such as amiodarone, dopamine or heparin), which could affect thyroid metabolism or result in interferences in laboratory measurements (2,3). Therefore, considering all the clinical and laboratory interferences in critically ill patients, thyroid function evaluation and NTIS diagnosis represent a challenge to physicians.

Although we do not fully understand the physiopathology of NTIS, we already know an essential part of its mechanism (4). NTIS is mainly related to decreased deiodinase type 1 (DIO1) activity, abnormal deiodinase type 3 (DIO3) production and thyroid axis suppression with an inappropriately normal thyroid-stimulating hormone (TSH) (4-7). These alterations promote tissue and systemic triiodothyronine (T3) drops associated with an increase in reverse T3 (rT3) in the presence of TSH value in the reference range and with normal or low concentrations of thyroxine (T4) (8). Even though thyroid hormone concentrations during healthy childhood and adulthood are different, the thyroid axis changes caused by NTIS in the newborn, child and adult are the same (3,9). The debate persists about whether NTIS involves an adaptative response or real hypothyroidism at the tissue level (10). The NTIS-related tissue decrease in T3 probably leads to indistinguishable gene expression repercussions similar to those observed in hypothyroid patients.

The unfavorable prognosis observed in low thyroid hormone concentrations is found in different clinical settings and study designs (11-18). Septic shock is a significant cause of death in intensive care units (ICUs) and is associated with NTIS in newborns, children, and adults (4,19,20).

Castro and cols. in an experimental model of septic shock, demonstrated systemic and tissue decreases in T4 and T3 (21). Taşcı and cols. also showed that sepsis progression was less severe in the hyperthyroid group and more severe in the hypothyroid group (22). The prevalence of NTIS in critically ill patients may vary from 27.5% to 38.7%, but it is even higher in cases of sepsis (19,23,24). In addition, this prevalence is probably underestimated. There are laboratorial difficulties in measuring thyroid hormones in such clinical scenarios, as medications cause interference, and the neuroendocrine response to stress dynamic evolution is also a confounding factor (25). Therefore, thyroid hormone concentrations with or without reverse T3 (rT3) measures might only lead us to the suspicion of NTIS (1).

We hypothesized that the circulating RNA measurement alterations might directly evaluate the hormonal action from the blood cells or could be an indirect reflection of the tissue thyroid hormone repercussion, which can be obtained in a less invasive form. In the blood, these RNA alterations can result from the canonical and noncanonical thyroid hormone action leading to changes in gene expression (26). On the other hand, the tissue microRNA (miR) expression can be transported through vesicles to circulation and affect mRNA production (27). To emphasize this point of view, the Translational Safety Biomarker Pipeline (TransBioLine) published a Letter of Intent (LOI) in 2020, which was accepted by the Food & Drug Administration (FDA), establishing that circulating miR can be used as a non-invasive tool for tissue and mechanism-specific diagnosis.

The adipose tissue is the main source of miR in the circulation, and we have long known that thyroid hormones affect the RNA expression in this tissue (27,28). Additionally, thyroid hormones, primarily T3, participate directly in metabolism by mediating the transcription of mitochondrial proteins (29,30).

Our study aims to identify differentially expressed genes (DEGs), focusing on mitochondrial genes in hypothyroid patients without NTIS and correlating with sepsis and septic shock patients. The concordant profile between hypothyroid and septic shock patients may provide new biomarkers for challenging NTIS scenarios.

## MATERIALS AND METHODS

### Population

This study was conducted in accordance with the ethical principles of the Declaration of Helsinki and was approved by the Institutional Review Board (number 665,331; CAAE: 30746814.4.0000.5511). The transcriptome hypothyroidism study participants attended the university outpatient clinic and signed informed consent forms.

### Blood samples, biochemical analysis, RNA extraction and cDNA synthesis from transcriptome hypothyroidism study

Venous blood samples were used for biochemical and RNA analyses. The blood for the total RNA analysis was collected and preserved with PAXgene blood RNA (Qiagen, NL, DE). TSH, free thyroxine (FT4), antithyroglobulin antibody (TgAb) and anti-thyroperoxidase antibody (TPOAb) analyses were performed with an Elecsys 2010 (Roche Diagnostics, IN, USA), following specific automated protocols for each test. The TSH reference values were 0.270-4.50 mU/L. The FT4 reference values were 0.93 to 1.70 ng/dL. The TgAb negative reference value was less than 115 IU/mL, and the TPOAb negative reference value was less than 34 IU/mL.

Total RNA was obtained from peripheral blood and extracted using the PAXgene Blood RNA Kit (Qiagen, NL, DE). The quantification of total RNA was performed on a Qubit Fluorometer 2.0 with its respective kit (Thermo Fisher Scientific, MA, USA). cDNA synthesis was performed using the SuperScript VILO Mastermix kit (Thermo Fisher Scientific, MA, USA) following the recommended protocol.

### Hypothyroidism transcriptome libraries

The libraries were constructed with four individuals for the healthy euthyroid control group (CTL) and four patients for the hypothyroid group (HT). HT patients have never been treated with levothyroxine. The CTL individuals have a stable and reference range TSH. In contrast, the HT group also had stable TSH above 10 mU/L. The eight libraries used in this study are available on GEO (https://www.ncbi.nlm.nih.gov/geo/, accession number: GSE176153).

The transcriptome libraries were constructed using Ion Proton System next-generation sequencing (Thermo Fisher Scientific, MA, USA) with Ion AmpliSeq Transcriptome Human Gene Expression Kit protocols.

### Bioinformatics workflow for transcriptome analysis

Transcriptome data analysis was performed using R Software version 2021.09.2 build 382 (31). The data were normalized using the trimmed mean of M-values (TMM), which uses the stable internal genes to establish the dispersion (32). The NOISeq package (version 2.38.0) was used to call the DEG (33). The analysis pipeline is available in Supplementary File 1. To produce the intersection data, we use the tool InteractiVenn (34).

The characterization of mitochondrial RNAs was performed by the Human MitoCarta 3.0 database (35). The Reactome database was used to analyze gene pathways in FunRich software version 3.1.3 (36,37).

### Critical illness GEO Datasets

Based on the high prevalence of NTIS on sepsis, we searched for NTIS databanks in GEO Datasets (https://www.ncbi.nlm.nih.gov/gds) and selected two datasets for analysis. One is in the adult scenario (GSE54514), and the other is in the children scenario (GSE26440). In GSE54514, we separated the analyses into two blocks: sepsis survivors versus control and sepsis nonsurvivors versus control. The control group comprised thirty-six individuals, the sepsis survivor group comprised ninety-six, and thirty-one individuals formed the sepsis group's nonsurvivors (38).

In GSE26440, we divided the analyses into two blocks: sepsis survivors versus control and sepsis nonsurvivors versus control. The control group consisted of thirty-two children, the sepsis survivor group comprised eighty-one children and seventeen children in the sepsis group's nonsurvivors (39).

### Bioinformatics workflow for microarray

All analyses were performed in R Software (31). The Limma package (version 3.50.0) was used to identify the differentially expressed genes in the microarrays. GEOquery (version 2.62.2) connected the chosen database with the software, and UMAP (version 0.2.8.8) was used to construct the array according to the selected datasets. The microarray analysis pipelines are available in Supplementary File 1. We considered differentially expressed transcripts with an FDR < 0.05. To produce the intersection data, we used the tool InteractiVenn (34).

The characterization of mitochondrial RNAs was performed by the Human MitoCarta 3.0 database (35). The Reactome database was used to analyze gene pathways in FunRich software version 3.1.3 (36,37).

### Statistical analysis

Data are mainly presented as median, percentiles, and maximum and minimum values. The Mann-Whitney test was used to perform the two-group analysis of the continuous variables. We used the ROC curve to calculate the area under the curve (AUC) and established the cutoff point by Youden's method. The categorical variables were analyzed by the chi-square test (χ2) with Fisher's exact test when necessary. Sensitivity, specificity, negative predictive value (NPV), and positive predictive value (PPV) were calculated based on the Galen and Gambino formula. We also estimated the prevalence (pretest probability) and accuracy to weigh the biomarker values in each dataset. Cohen's kappa was used to avoid errors induced by missing data, and positive and negative likelihood ratios were calculated because prevalence does not influence them. Cox regression and the Kaplan-Meier method were used for the survival analysis. A p-value < 0.05 was considered significant. IBM SPSS Statistics for Windows, Version 26.0, from IBM Corp., released in 2019 (Armonk, NY, USA), was used to analyze the data.

## RESULTS

### Transcriptome analysis

**Figure 1:**
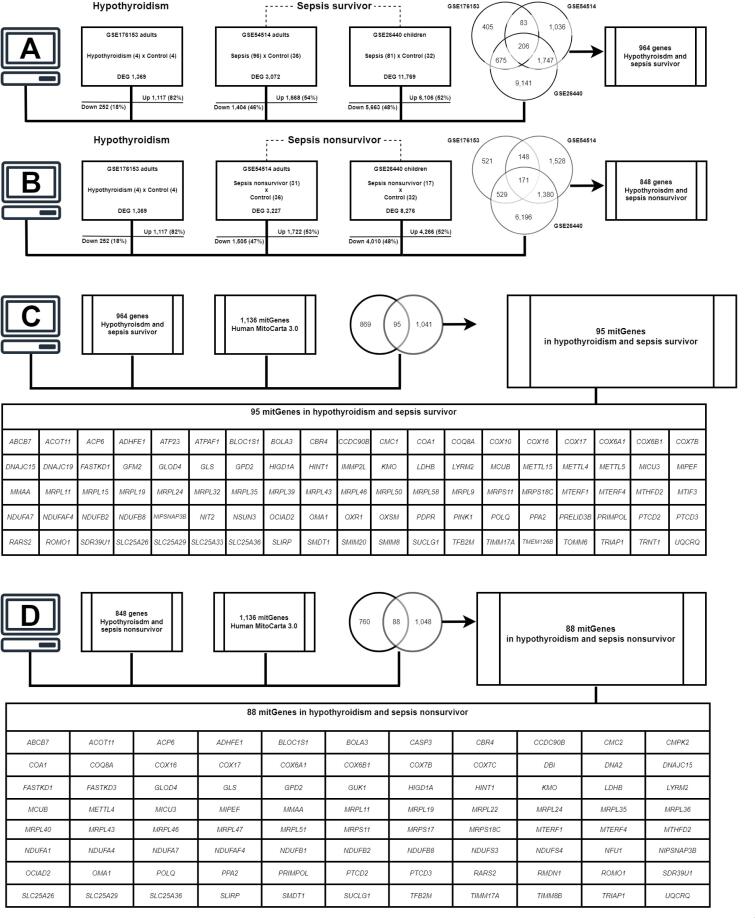
Shared genes between hypothyroidism and adult and pediatric sepsis survivors and nonsurvivors. (**A**) Differentially expressed gene (DEG) workflow between hypothyroidism and sepsis survivors in adults and children. (**B**) DEG workflow between hypothyroidism and sepsis nonsurvivors in both scenarios. (**C**) The mitGenes when comparing shared genes between hypothyroidism and sepsis survivors. (**D**) The mitGenes when comparing hypothyroidism and sepsis nonsurvivor shared genes.

The hypothyroidism scenario was identified on the GSE176153 dataset, which compared healthy participants and hypothyroidism patients. We included at least one male in each group to avoid a strong sex influence. The clinical and laboratory parameters are shown in Table 1. The analysis revealed 1,369 DEGs regulated by thyroid hormones in peripheral blood, represented in the first box from Figure 1A, B.

**Table 1:** Clinical and laboratory parameters from the hypothyroidism transcriptome (GSE176153)

	Control	Hypothyroidism	p value
Sex (male/female)	1/3	1/3	1.00
Age (years)	38 (36-40)	44.5 (34-52)	0.56
TSH (μUI/mL)	2.35 (1.3-4.2)	82.3 (58.6->100)	0.02
FT4 (ng/dL)	1.22 (1.1-1.3)	0.2 (0.1-0.5)	0.02
TgAb (UI/mL)	<10	766 (<10-1195)	0.09
TPOAb (UI/mL)	9.3 (<5-32.4)	198 (<5-515)	0.24

### Microarray analysis

The analysis of the GSE54514 dataset formed by the sepsis survivor and control (adults) groups revealed 3,072 DEGs in peripheral blood, represented in the second box in Figure 1A. The analysis of the sepsis nonsurvivor and control (adults) groups revealed 3,227 DEGs in peripheral blood, as described in the second box in Figure 1B.

The analysis of the GSE26440 dataset formed by the sepsis survivor and control (children) groups revealed 11,769 DEGs in peripheral blood, represented in the third box in Figure 1A. The analysis of the sepsis nonsurvivor and control groups (children) revealed 8,276 DEGs in peripheral blood, as described in the third box in Figure 1B.

### Comparison of the DEGs in sepsis

#### Hypothyroidism versus sepsis survivors

Comparing hypothyroidism (GSE176153) versus sepsis survivors in GSE54514 and GSE26440, we found 964 shared DEGs, as shown in Supplementary List 1 and Figure 1A.

#### Hypothyroidism versus sepsis nonsurvivors

Comparing hypothyroidism (GSE176153) versus sepsis nonsurvivors (GSE54514 and GSE26440), we found 848 shared DEGs, as shown in Supplementary List 1 and Figure 1B.

### Mitochondrial genes

Intersecting the 964 DEGs present in hypothyroidism and sepsis survivors with the list of 1136 human mitochondrial genes (mitGenes) in MitoCarta 3.0, we found 95 mitGenes (10%) in this scenario, as shown in Supplementary List 2 and Figure 1C.

Intersecting the 848 DEGs present in hypothyroidism and sepsis nonsurvivors with the 1136 human mitochondrial genes list (mitGenes) MitoCarta 3.0, we found 88 mitGenes (10%) in this scenario, as shown in Supplementary List 2 and Figure 1D.

#### Agreement in the increased or decreased expression levels of mitGenes in the analyzed scenarios

We looked at the mitGenes concordant logarithmic fold change (logFC) between hypothyroidism, sepsis survivors and sepsis nonsurvivors. From 964 shared genes between hypothyroidism and sepsis survivors, we found 95 mitGenes. All 95 mitGenes were present in hypothyroidism; 92 (97%) were overexpressed, and only 3 (3%) were underexpressed. In the adult sepsis survivor group, we found 26 of the 95 mitGenes (27%); 11 of them were overexpressed (42%) and 15 mitGenes were underexpressed (58%). In the child sepsis survivor group, we found 85 of the 95 mitGenes (90%); 13 (15%) were overexpressed and 72 (85%) were underexpressed.

From 848 shared genes between hypothyroidism and sepsis nonsurvivors, we found 88 mitGenes. They all appeared in hypothyroidism, with 85 (97%) mitGenes with increased expression and 3 (3%) mitGenes with decreased expression. In the adult sepsis nonsurvivor group, we found 48 of the 85 mitGenes; 43 (90%) were overexpressed and 5 (10%) were underexpressed. In the child septic nonsurvivor group, we found 64 of the 85 mitGenes; 25 (39%) were overexpressed and 39 (61%) were underexpressed.

We observed concordant increased expression of the *BLOC1S1* and *ROMO1* mitGenes in the hypothyroidism and sepsis survivor scenarios (adult and child). However, we did not find underexpressed mitGenes between these three scenarios. In addition, *COX6A1*, *COX7B*, *DBI*, *MRPL22*, *MRPL51*, *MRPS18C*, *NDUFA4*, *POLQ*, *SLIRP*, *TIMM8B*, *UQCRQ*, and also *BLOC1S1* and *ROMO1* mitGenes had concordant gains of expression in the hypothyroidism and nonsurvivor sepsis scenarios. However, we did not find any mitGenes with loss of expression between these three scenarios.

**Figure 2:**
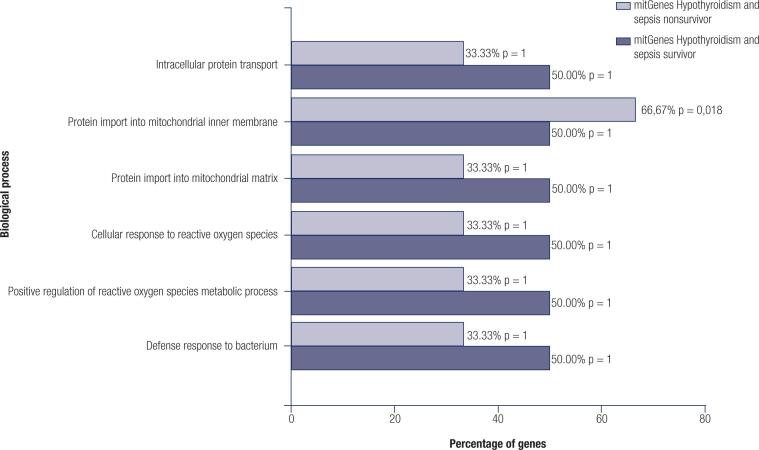
Biological pathways identified between the groups. Shared hypothyroidism mitochondrial genes and biological processes present in sepsis survivors and nonsurvivors are shown. The percentage of genes was calculated from the number of genes available in the database.

As we were interested in genes related to the ATP production mechanism, we considered the biological processes of *BLOC1S1*, *ROMO1*, *SLIRP* and *TIMM8B* mitGenes shared between all scenarios (hypothyroidism, sepsis survivors and nonsurvivors) and constructed a plot, as shown in Figure 2.

**Figure 3:**
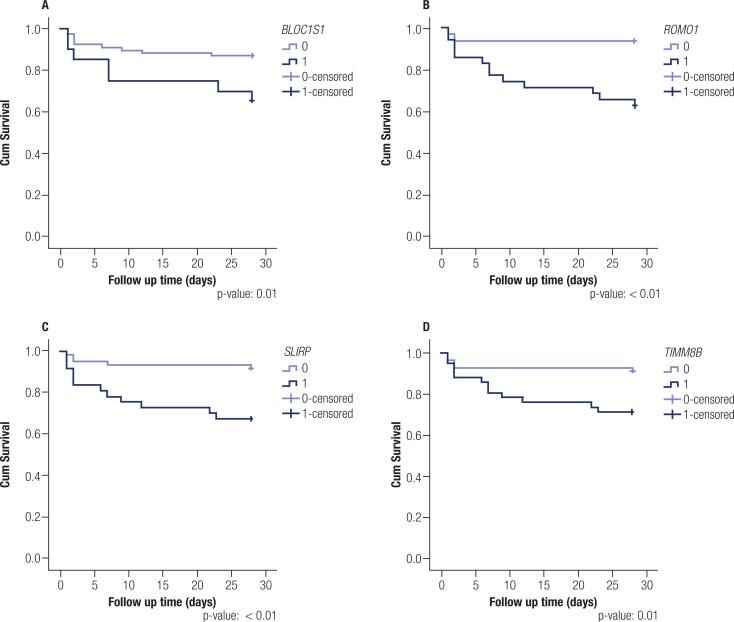
Survival curves of children with sepsis. The selected genes were shared between hypothyroidism, sepsis survivors and nonsurvivors scenarios and show the capability to distinguish sepsis survivors and nonsurvivors in children. The follow-up time was tracked for 28 days after admission by Wong et al. A- *BLOC1S1*; B- *ROMO1*; C- *SLIRP* and D- *TIMM8B*.

We evaluated the expression levels of four mitochondrial nonsurvivor genes that appeared in the three datasets and exhibited concordant expression. *ROMO1* and *TIMM8B* showed higher AUCs, specificities, and accuracies in adults. *ROMO1* and *SLIRP* showed higher AUCs, sensitivities, specificities, and accuracies in children (Table 2). Unfortunately, we could only construct children's survival curves and hazard ratios (GSE26440) (Figure 3 and Table 3). The follow-up time was tracked for 28 days after admission (40). Moreover, the selected mitochondrial genes showed the ability to distinguish sepsis survivors from nonsurvivors, as illustrated in Figure 3.

**Table 2:** Analysis summary of the 4 differentially expressed mitochondrial genes from sepsis nonsurvivors. The area under the curve (AUC), chi-square, chi-square p value, sensibility, specificity, positive and negative predictive values, accuracy, positive and negative likelihood ratios, Cohen's kappa and the kappa p value of each gene were calculated based on the gene expression from sepsis survivors and nonsurvivors in dataset GSE54514 from adults and GSE26440 from children. GSE54514 showed a prevalence of mortality of 24.41%, and GSE26440 showed a prevalence of 14.65%. A p value < 0.05 was considered significant

GSE54514 Adult Dataset				
Gene symbol	*BLOC1S1*	*ROMO1*	*SLIRP*	*TIMM8B*
AUC	0.62	0.71	0.64	0.80
Chi-square	6.39	22.03	8.81	32.95
Chi-square (p value)	0.01	<0.01	0.003	<0.01
Sensibility	0.65	0.65	0.77	0.74
Specificity	0.61	0.8	0.53	0.81
Positive predictive value	0.35	0.51	0.35	0.56
Negative predictive value	0.84	0.88	0.88	0.91
Accuracy	0.62	0.76	0.59	0.8
Cohen's kappa	0.20	0.41	0.22	0.50
Cohen's kappa (p value)	0.01	<0.01	<0.01	<0.01
Positive likelihood ratio	2.23	3.70	2.15	4.43
Negative likelihood ratio	0.25	0.16	0.20	0.12
**GSE26440 Pediatric Dataset**				
**Gene symbol**	***BLOC1S1***	***ROMO1***	***SLIRP***	***TIMM8B***
AUC	0.66	0.80	0.72	0.70
Chi-square	8.00	17.28	9.59	7.47
Chi-square (p value)	0.01	<0.01	<0.01	<0.01
Sensibility	0.59	0.76	0.71	0.71
Specificity	0.13	0.75	0.69	0.65
Positive predictive value	0.1	0.34	0.28	0.26
Negative predictive value	0.65	0.95	0.93	0.93
Accuracy	0.2	0.75	0.69	0.66
Cohen's kappa	0.26	0.34	0.24	0.20
Cohen's kappa (p value)	<0.01	<0.01	<0.01	<0.01
Positive likelihood ratio	0.81	3.79	3.00	2.69
Negative likelihood ratio	1.77	0.07	0.10	0.11

**Table 3:** Hazard ratios of nonsurvival for concordant expression genes between hypothyroidism and sepsis in children. Nonsurvival ratios with 95% confidence intervals (CIs) of *BLOC1S1*, *ROMO1*, *SLIRP* and *TIMM8B* expression in septic children (GSE26440)

Gene	Hazard Ratio	95% CI	p value
*BLOC1S1*	1.36	0.96-1.91	0.08
*ROMO1*	2.75	1.56-4.72	<0.01
*SLIRP*	1.65	1.21-2.25	<0.01
*TIMM8B*	1.85	1.15-2.98	0.01

## DISCUSSION

NTIS is still considered an adaptative response in critically ill patients. To date, treatment with levothyroxine or liothyronine is not recommended in NTIS (8). Nevertheless, NTIS occurred in 62.9% of critically ill children and was an independent predictor of mortality (41). It has long been known that a low serum T4 is associated with an increased probability of death (42). In critically ill patients and those with liver failure, NTIS was observed in 67.12% and was associated with a higher mortality rate than in those without the syndrome (43). In sepsis, NTIS was associated with mortality, and a low total T3, free T3, or the combination of low T3 with low T4 are predictors of mortality (44).

Bedside evaluation represents a real challenge for critically ill patients, especially those with a severe infectious disease such as sepsis, because multiple direct and indirect dysfunctions occur at the molecular and cellular levels (38). As Todd and cols. reported, part of this complexity may be related to NTIS (45). We identified a similar DEG in the blood pattern between hypothyroidism and septic patients, even with different datasets constructed with different methodologies. Our study identified that 964 (70.42%) and 848 (61.94%) of our hypothyroidism DEGs were shared with sepsis survivors and nonsurvivors, respectively.

Based on the thyroid hormone actions, as expected, some of our identified genes were mitochondrial types. Mitochondrial genes are primarily responsible for the production of ATP. In our study, 90% of mitochondrial genes are overexpressed in adult sepsis nonsurvivors. On the other hand, only 39% were overexpressed in children nonsurvivors.

Although critical care survival runs differently among adult and child populations, our main intention was to identify the similarities in the NTIS mechanism in those two populations (46). However, the challenge in comparing those two populations was many because they have distinct backgrounds. These differences, for example, are the epidemiological profile, the previously undiagnosed illnesses, the disease that progresses to sepsis and the different clinical responses (47).

Although we are hunting for similarities, not differences, the difference between adult and children populations deserves more consideration. This apparent disagreement could have two explanations, one physiological and another analytical. The physiological explanation is that some genes involved in children's growth and development could be already turned on (48). Consequently, these growth and development genes may affect our analytic strategy. It happens because we used the TMM normalization strategy, and the stable genes through the samples are used to calculate the normalization factor (32). So different populations with different genes composing the baseline alter the normalization factor and influence the significance of some genes.

Furthermore, some mitGenes with a concordant expression gain related to ATP production mechanism were shared between hypothyroidism and sepsis: *SLIRP* and *TIMM8B* appeared in sepsis nonsurvivor scenarios; *ROMO1* and *BLOC1S1* were present in both survivor and nonsurvivor scenarios. These specific mitGenes can represent NTIS biomarkers in nonsurvivors (*SLIRP* and *TIMM8B*).

Mitochondrial functions are necessary for ATP production and control of apoptosis mechanisms (49). Long-term mitochondrial function and genes are associated with thyroid hormone influence (50). Thyroid hormones regulate critical biological processes, such as energy consumption, thermogenesis, cell development and growth (51). Sepsis can also interfere with mitochondrial functions and cause damage to the mitochondrial electron transport chain (49). Due to the inflammatory response, the increase in reactive oxygen species (ROS) leads to a change in mitochondria, causing a drop in ATP levels (52,53).

In hyperglycemic animal cardiomyocyte hypertrophy cells, *TIMM8B* was overexpressed in the colonic mucosa and myocardium (54,55). In addition, hyperglycemia is seen in patients with sepsis (56). In accordance with our results, *TIMM8B* was up-regulated in hypothyroidism and only in nonsurvivors patients. Also, the overexpression showed an increased risk of death outcome, and *TIMM8B* could distinguish who survivor or not in children.

The mitGene *SLIRP* encodes a protein with a stabilizing function of ribosomal mRNA strands, which protects them from degradation, prevents abnormalities in the translation process, and plays a role in mitochondrial quality control between untranslated transcripts (57). The action of the mitGene *SLIRP* guarantees a fundamental role in the maintenance of translations of transcripts that encode the subunits of proteins linked to oxidative phosphorylation, the primary cellular pathway for obtaining ATP (58). In our study, *SLIRP* was up-regulated in hypothyroidism and only in nonsurvivors, possibly demonstrating the attempt to stabilize the mitochondria by maintaining adequate protein synthesis levels. In this case, principally, proteins are linked to the production of ATP, which will be essential in mitochondrial, cellular and tissue homeostasis in hypothyroidism and the fight against sepsis. This gene was also able to identify who survived, and the gene overexpression showed an increased risk of death in children with sepsis.

The MitGene *BLOC1S1*, also known as *GCN5L1*, has a critical protein-coding role with a homologous function of the acetyltransferase enzyme. This protein participates in acetyl-CoA binding, modulating the acetylation of electron transport chain proteins, whose final impact is directly linked to mitochondrial oxygen consumption and ATP levels (59). The revealed increase in its expression in hypothyroidism and sepsis contributes to energy maintenance in these diseases.

*ROMO1* encodes a protein present in the mitochondrial membrane and is responsible for the increase in the production of reactive oxygen species. This same protein has antimicrobial activity against several bacterial species. This gene is already a potential biomarker in diagnosing and predicting many diseases, including prostate and lung cancers, inflammation and oxidative stress in chronic obstructive pulmonary disease (60,61). The antimicrobial action already justifies the increase in expression in patients with sepsis. The oxidative stress produced during sepsis, resulting from the increase in reactive oxygen species, also reinforces the increase in the expression of mitGene. This possible condition is strengthened when we see an increase in the expression of the mitGene *ROMO1* in hypothyroidism. In our study, *BLOC1S1* and also *ROMO1* were up-regulated in all the scenarios: in hypothyroidism, survivors and non-survivors. Despite that, *BLOC1S1* and *ROMO1* could distinguish the children who survived or not. However, only *ROMO1* showed an increased risk of death outcome in children with sepsis.

The decrease in metabolic expenditure would be favorable for preserving life in a critical care situation. However, reducing muscle strength, especially the respiratory or cardiac muscle, contributes to poor patient prognosis. Additionally, diaphragm weakness increases the mortality rate in critically ill patients (62). An experimental sepsis model with NTIS showed that decreased thyroid hormones led to severe changes in mitochondrial physiology in the diaphragm (63). In sepsis, skeletal musculature deiodinase activity can improve muscle repair, injury or muscular atrophy (51).

In adult sepsis, *BLOC1S1*, *ROMO1*, *SLIRP* and *TIMM8B* showed excellent ability to identify nonsurvivor samples. We observed the same results in children, except for the mitGene *BLOC1S1*. *ROMO1*, *SLIRP* and *TIMM8B* led to an elevated risk of nonsurvivor outcomes in children.

Our study has some drawbacks, as the datasets used were not designed to look for NTIS, and the thyroid hormone concentrations are unavailable. In addition, the different RNA detection methodologies and bioinformatic strategies represent another fragility, especially for correctly defining lost or gained expression. Furthermore, circulating RNA is mainly influenced by thyroid receptor alpha; in other words, this RNA profile reflects only a part of the whole scenario (64). Also, sepsis databases were used in different populations, adults and children, and unfortunately, the information about adults’ follow-up time was unavailable. However, although the pattern of adults and children with sepsis is not precisely the same, we noticed that some genes found in our study are common in both. As a final point, we found a similar pattern between hypothyroid patients and those with sepsis, which could be the molecular fingerprint of NTIS. We also identified potential candidate genes for a biomarker panel of nonsurvivors patients.

Additionally, some genes could distinguish sepsis survivors and nonsurvivors and showed an increased risk of developing death outcomes in children. Therefore, we theorize that, in this scenario, after identifying the nonsurvivors’ expression pattern, the treatment with levothyroxine in the correctly selected group could improve survival. However, more research is needed to evaluate these genes in a well-designed study to control for confounders.

## Supplementary Pipeline 1.

Pipeline analysis of databases: GSE176153, GSE54514 and GSE26440

### GSE176153 - Hypothyroidism

if(!require("BiocManager",quietly=TRUE))

install.packages("BiocManager")

BiocManager::install("NOISeq")

BiocManager::install("org.Hs.eg.db")

BiocManager::install("edgeR")

BiocManager::install("clusterProfiler")

setwd("Select your directory")

require(NOISeq)

require(org.Hs.eg.db)

require(edgeR)

require(clusterProfiler)

df<-read.table("GSE176153.txt",header=TRUE,row.names=1)

design<-as.factor(rep(c("1","2"),each=4))

depth<-list(sum(df$CTL1),sum(df$CTL2),sum(df$CTL3),

sum(df$CTL4),sum(df$HT1),sum(df$HT2),sum(df$HT3),sum(df$HT4))

bol<-filterByExpr(df,design)

df<-df[bol,]

rm(bol)

df<-tmm(df)

myfactors<-data.frame(Thyroid = c("1","1","1","1","2","2",

"2","2"),ThyroidRun=c("1_1","1_1","1_1","1_1","2_2","2_2",

"2_2","2_2"),Run=c(rep("R2",4),rep("R2",4)))

mydata<-readData(data=df,factors=myfactors)

mynoiseq<-noiseqbio(mydata,norm="n",factor="Thyroid",

filter=3,a0per=0.9,depth=c(depth[[1]],depth[[2]],

depth[[3]],depth[[4]],depth[[5]],depth[[6]],depth[[7]],

depth[[8]]))

mynoiseq.deg<-degenes(mynoiseq,q=.95)

mynoiseq.deg<-mynoiseq.deg %>% filter(log2FC> 0.5|log2FC< -0.5)

mynoiseq.deg$symbol=mapIds(org.Hs.eg.db,keys=row.names

(mynoiseq.deg),column="SYMBOL",keytype="REFSEQ",multiVals="first")

write.csv(mynoiseq.deg,file="Output DGE GSE176153.csv")

### GSE54514 – Sepsis survivor adults

if (!require("BiocManager", quietly = TRUE))

install.packages("BiocManager")

BiocManager::install("GEOquery")

BiocManager::install("limma")

install.packages("umap")

setwd("Select your directory")

require(GEOquery)

require(limma)

require(umap)

gset <- getGEO("GSE54514", GSEMatrix =TRUE, AnnotGPL=TRUE)

if (length(gset) > 1) idx <- grep("GPL6947", attr(gset, "names")) else idx <- 1

gset <- gset[[idx]]

fvarLabels(gset) <- make.names(fvarLabels(gset))

gsms <- paste0("000000000000000000XXXXXXXXXXXXXXXXXXXXXXXXXXXXXXX1",

"11111111111111111111111111111111111111111111111111",

"11111111111111111111111111111111111111111111100000",

"0000000000000")

sml <- strsplit(gsms, split="")[[1]]

sel <- which(sml != "X")

sml <- sml[sel]

gset <- gset[ ,sel]

ex <- exprs(gset)

qx <- as.numeric(quantile(ex, c(0., 0.25, 0.5, 0.75, 0.99, 1.0), na.rm=T))

LogC <- (qx[5] > 100) ||

(qx[6]-qx[1] > 50 && qx[2] > 0)

if (LogC) { ex[which(ex <= 0)] <- NaN

exprs(gset) <- log2(ex) }

gs <- factor(sml)

groups <- make.names(c("Sepsis survivor","Control"))

levels(gs) <- groups

gset$group <- gs

design <- model.matrix(~group + 0, gset)

colnames(design) <- levels(gs)

fit <- lmFit(gset, design)

cts <- paste(groups[1], groups[2], sep="-")

cont.matrix <- makeContrasts(contrasts=cts, levels=design)

fit2 <- contrasts.fit(fit, cont.matrix)

fit2 <- eBayes(fit2, 0.01)

tT <- topTable(fit2, adjust="fdr", sort.by="B", number=Inf)

tT <- subset(tT, select=c("ID","adj.P.Val","P.Value","t","B","logFC","Gene.symbol","Gene.title"))

write.table(tT, file=stdout(), row.names=F, sep="\t")

tT=subset(tT, adj.P.Val<0.05)

write.csv(tT,file="Output DGE GSE54514 Sepsis survivor.csv")

### GSE54514 – Sepsis nonsurvivor adults

setwd("Select your directory")

require(GEOquery)

require(limma)

require(umap)

gset <- getGEO("GSE54514", GSEMatrix =TRUE, AnnotGPL=TRUE)

if (length(gset) > 1) idx <- grep("GPL6947", attr(gset, "names")) else idx <- 1

gset <- gset[[idx]]

fvarLabels(gset) <- make.names(fvarLabels(gset))

gsms <- paste0("0000000000000000001111111111111111111111111111111X",

"XXXXXXXXXXXXXXXXXXXXXXXXXXXXXXXXXXXXXXXXXXXXXXXXXX",

"XXXXXXXXXXXXXXXXXXXXXXXXXXXXXXXXXXXXXXXXXXXXX00000",

"0000000000000")

sml <- strsplit(gsms, split="")[[1]]

sel <- which(sml != "X")

sml <- sml[sel]

gset <- gset[ ,sel]

ex <- exprs(gset)

qx <- as.numeric(quantile(ex, c(0., 0.25, 0.5, 0.75, 0.99, 1.0), na.rm=T))

LogC <- (qx[5] > 100) ||

(qx[6]-qx[1] > 50 && qx[2] > 0)

if (LogC) { ex[which(ex <= 0)] <- NaN

exprs(gset) <- log2(ex) }

gs <- factor(sml)

groups <- make.names(c("Sepsis nonsurvivor","Control"))

levels(gs) <- groups

gset$group <- gs

design <- model.matrix(~group + 0, gset)

colnames(design) <- levels(gs)

fit <- lmFit(gset, design)

cts <- paste(groups[1], groups[2], sep="-")

cont.matrix <- makeContrasts(contrasts=cts, levels=design)

fit2 <- contrasts.fit(fit, cont.matrix)

fit2 <- eBayes(fit2, 0.01)

tT <- topTable(fit2, adjust="fdr", sort.by="B", number=Inf)

tT <- subset(tT, select=c("ID","adj.P.Val","P.Value","t","B","logFC","Gene.symbol","Gene.title"))

write.table(tT, file=stdout(), row.names=F, sep="\t")

tT=subset(tT, adj.P.Val<0.05)

write.csv(tT,file="Output DGE GSE54514 Sepsis nonsurvivor.csv")

### GSE26440 – Sepsis survivor children

setwd("Select your directory")

require(GEOquery)

require(limma)

require(umap)

gset <- getGEO("GSE26440", GSEMatrix =TRUE, AnnotGPL=TRUE)

if (length(gset) > 1) idx <- grep("GPL570", attr(gset, "names")) else idx <- 1

gset <- gset[[idx]]

gsms <- paste0("X111111111111111111111111X11X1111111X1XX00011XX000",

"00111X1111111X11111XX11111110010000X00001011110000",

"00000111X000101X11X111111111X1")

sml <- strsplit(gsms, split="")[[1]]

sel <- which(sml != "X")

sml <- sml[sel]

gset <- gset[ ,sel]

ex <- exprs(gset)

qx <- as.numeric(quantile(ex, c(0., 0.25, 0.5, 0.75, 0.99, 1.0), na.rm=T))

LogC <- (qx[5] > 100) ||

(qx[6]-qx[1] > 50 && qx[2] > 0)

if (LogC) { ex[which(ex <= 0)] <- NaN

exprs(gset) <- log2(ex) }

gs <- factor(sml)

groups <- make.names(c("Sepsis survivor","Control"))

levels(gs) <- groups

gset$group <- gs

design <- model.matrix(~group + 0, gset)

colnames(design) <- levels(gs)

fit <- lmFit(gset, design)

cts <- paste(groups[1], groups[2], sep="-")

cont.matrix <- makeContrasts(contrasts=cts, levels=design)

fit2 <- contrasts.fit(fit, cont.matrix)

fit2 <- eBayes(fit2, 0.01)

tT <- topTable(fit2, adjust="fdr", sort.by="B", number=Inf)

tT <- subset(tT, select=c("ID","adj.P.Val","P.Value","t","B","logFC","Gene.symbol","Gene.title"))

write.table(tT, file=stdout(), row.names=F, sep="\t")

tT=subset(tT, adj.P.Val<0.05)

write.csv(tT,file="Output DGE GSE26440 Children sepsis survivor.csv")

### GSE26440 – Sepsis nonsurvivor children

setwd("Select your directory")

require(GEOquery)

require(limma)

require(umap)

gset <- getGEO("GSE26440", GSEMatrix =TRUE, AnnotGPL=TRUE)

if (length(gset) > 1) idx <- grep("GPL570", attr(gset, "names")) else idx <- 1

gset <- gset[[idx]]

fvarLabels(gset) <- make.names(fvarLabels(gset))

gsms <- paste0("1XXXXXXXXXXXXXXXXXXXXXXXX1XX1XXXXXXX1X11000XX11000",

"00XXX1XXXXXXX1XXXXX11XXXXXXX00X000010000X0XXXX0000",

"00000XXX1000X0X1XX1XXXXXXXXX1X")

sml <- strsplit(gsms, split="")[[1]]

sel <- which(sml != "X")

sml <- sml[sel]

gset <- gset[ ,sel]

ex <- exprs(gset)

qx <- as.numeric(quantile(ex, c(0., 0.25, 0.5, 0.75, 0.99, 1.0), na.rm=T))

LogC <- (qx[5] > 100) ||

(qx[6]-qx[1] > 50 && qx[2] > 0)

if (LogC) { ex[which(ex <= 0)] <- NaN

exprs(gset) <- log2(ex) }

gs <- factor(sml)

groups <- make.names(c("Sepsis nonsurvivor","Control"))

levels(gs) <- groups

gset$group <- gs

design <- model.matrix(~group + 0, gset)

colnames(design) <- levels(gs)

fit <- lmFit(gset, design)

cts <- paste(groups[1], groups[2], sep="-")

cont.matrix <- makeContrasts(contrasts=cts, levels=design)

fit2 <- contrasts.fit(fit, cont.matrix)

fit2 <- eBayes(fit2, 0.01)

tT <- topTable(fit2, adjust="fdr", sort.by="B", number=Inf)

tT <- subset(tT, select=c("ID","adj.P.Val","P.Value","t","B","logFC","Gene.symbol","Gene.title"))

write.table(tT, file=stdout(), row.names=F, sep="\t")

tT=subset(tT, adj.P.Val<0.05)

write.csv(tT,file="Output DGE GSE26440 Children sepsis nonsurvivor.csv")

## Supplementary list 1.

Differentially expressed genes (DEGs) between comparisons of hypothyroidism, sepsis survivors and sepsis nonsurvivors groups[Table t4]

**Table t4:**

DEG in hypothyroidism and sepsis survivor, n = 964	DEG in hypothyroidism and sepsis nonsurvivor, n = 848
*ABCA5*	*ABCA5*
*ABCB1*	*ABCB1*
*ABCB7*	*ABCB7*
*ABCC2*	*ABI1*
*ABI1*	*ACOT11*
*ABRACL*	*ACP1*
*ACAT2*	*ACP6*
*ACOT11*	*ACTN1*
*ACP1*	*ACTN4*
*ACP6*	*ACTR10*
*ACTN1*	*ADAMTS5*
*ACTN4*	*ADAP1*
*ADAMTS5*	*ADD1*
*ADAP1*	*ADGRA2*
*ADH5*	*ADH5*
*ADHFE1*	*ADHFE1*
*ADM*	*ADM*
*ADRB2*	*ADRB2*
*AIM2*	*AFDN*
*AKAP7*	*AIM2*
*AKT3*	*AKAP7*
*ALG6*	*AKT3*
*ALOX15*	*ALOX15*
*ANAPC4*	*AMPD2*
*ANKMY2*	*ANK1*
*ANKRA2*	*ANKMY2*
*ANKRD12*	*ANKRA2*
*ANKRD42*	*ANKRD12*
*ANKS1A*	*ANKS1A*
*ANXA1*	*ANXA1*
*ANXA11*	*ANXA11*
*AP3S1*	*AP3S1*
*APLF*	*APLP2*
*APLP2*	*APOBEC3G*
*APOBEC3G*	*APOBR*
*APOBR*	*ARF3*
*ARAP2*	*ARF4*
*ARF3*	*ARGLU1*
*ARF4*	*ARHGAP24*
*ARGLU1*	*ARHGAP4*
*ARHGAP24*	*ARL1*
*ARID5B*	*ARMC1*
*ARL1*	*ARPC1B*
*ARMC1*	*ARSB*
*ARMC8*	*ARV1*
*ARPC1B*	*ASB3*
*ARSB*	*ASCC2*
*ARV1*	*ASTE1*
*ASB3*	*ATAD2*
*ASCC2*	*ATG12*
*ASTE1*	*ATG4C*
*ATAD2*	*ATP10D*
*ATG12*	*ATP11B*
*ATG4C*	*ATP11C*
*ATP10D*	*ATP6V0A1*
*ATP11B*	*ATP6V0C*
*ATP11C*	*ATP6V0D1*
*ATP23*	*ATP6V1C1*
*ATP6V0A1*	*ATP6V1D*
*ATP6V0C*	*ATP6V1E1*
*ATP6V0D1*	*ATP6V1G1*
*ATP6V1C1*	*ATP8A1*
*ATP6V1D*	*B3GNT2*
*ATP6V1E1*	*BACE2*
*ATP6V1G1*	*BANK1*
*ATP8A1*	*BAZ2A*
*ATPAF1*	*BBIP1*
*B3GNT2*	*BBS9*
*BACE2*	*BBX*
*BANK1*	*BCAP31*
*BAZ2A*	*BCCIP*
*BBIP1*	*BDP1*
*BBS9*	*BEND4*
*BBX*	*BET1*
*BCAP29*	*BICD2*
*BCCIP*	*BLK*
*BDH2*	*BLOC1S1*
*BDP1*	*BLOC1S2*
*BEND4*	*BOLA2*
*BET1*	*BOLA3*
*BICD2*	*BRI3BP*
*BLK*	*BTAF1*
*BLOC1S1*	*BTF3*
*BOLA2*	*BTLA*
*BOLA3*	*BTN3A2*
*BORA*	*BTN3A3*
*BRI3BP*	*C12orf57*
*BTAF1*	*C12orf75*
*BTF3*	*C15orf39*
*BTLA*	*C1GALT1C1*
*BTN3A2*	*C1orf21*
*BTN3A3*	*C21orf91*
*C12orf57*	*CAMKK2*
*C12orf75*	*CAMP*
*C15orf39*	*CANT1*
*C18orf21*	*CAPN2*
*C1GALT1*	*CAPN7*
*C1GALT1C1*	*CAPNS2*
*C1orf21*	*CARD16*
*C21orf91*	*CARNMT1*
*CA1*	*CASK*
*CAMKK2*	*CASP3*
*CAMP*	*CBFA2T3*
*CANT1*	*CBR4*
*CAPN2*	*CCDC112*
*CAPN7*	*CCDC134*
*CAPNS2*	*CCDC141*
*CARD16*	*CCDC146*
*CARNMT1*	*CCDC15*
*CASK*	*CCDC66*
*CBR4*	*CCDC7*
*CBX3*	*CCDC82*
*CCDC134*	*CCDC90B*
*CCDC141*	*CCDC91*
*CCDC146*	*CCL5*
*CCDC15*	*CCND3*
*CCDC66*	*CCNJL*
*CCDC7*	*CCSER2*
*CCDC82*	*CD14*
*CCDC90B*	*CD160*
*CCDC91*	*CD180*
*CCL5*	*CD19*
*CCNC*	*CD1C*
*CCND3*	*CD200*
*CCNJL*	*CD244*
*CCNL1*	*CD300LB*
*CCSER2*	*CD3D*
*CD14*	*CD3G*
*CD160*	*CD47*
*CD180*	*CD52*
*CD19*	*CD53*
*CD1C*	*CD79A*
*CD200*	*CD79B*
*CD226*	*CD84*
*CD244*	*CD96*
*CD33*	*CDC42*
*CD3D*	*CDC5L*
*CD3G*	*CDK17*
*CD47*	*CDK18*
*CD52*	*CDKN2C*
*CD53*	*CDKN3*
*CD69*	*CEBPE*
*CD79A*	*CEP120*
*CD79B*	*CEP78*
*CD84*	*CETN2*
*CD96*	*CFAP44*
*CDC42*	*CHD3*
*CDC5L*	*CHI3L2*
*CDK17*	*CHML*
*CDK18*	*CKS2*
*CDKAL1*	*CLDND1*
*CDKN2C*	*CLEC2B*
*CDKN3*	*CLEC2D*
*CEBPE*	*CLTC*
*CENPP*	*CMC2*
*CEP120*	*CMPK2*
*CEP78*	*CNGA1*
*CETN2*	*CNOT7*
*CFAP44*	*CNTLN*
*CHD3*	*COA1*
*CHI3L2*	*COBLL1*
*CHML*	*COCH*
*CHORDC1*	*COL4A3*
*CKS2*	*COPS2*
*CLCN3*	*COPS4*
*CLDND1*	*COPS8*
*CLEC1A*	*COQ8A*
*CLEC2B*	*COX16*
*CLEC2D*	*COX17*
*CLIC2*	*COX6A1*
*CLK1*	*COX6B1*
*CLTC*	*COX7B*
*CMC1*	*COX7C*
*CMSS1*	*CPSF7*
*CNGA1*	*CPVL*
*CNOT7*	*CR2*
*CNOT9*	*CREBRF*
*CNTLN*	*CREBZF*
*COA1*	*CRTAM*
*COBLL1*	*CSK*
*COL7A1*	*CSNK1G2*
*COMMD3*	*CTNNA1*
*COPS4*	*CTSA*
*COPS8*	*CTSW*
*COQ8A*	*CUZD1*
*CORO1A*	*CWF19L2*
*COX10*	*CXorf65*
*COX16*	*CYFIP2*
*COX17*	*DBI*
*COX6A1*	*DCAF12*
*COX6B1*	*DCAF13*
*COX7B*	*DCP2*
*CPVL*	*DDA1*
*CR2*	*DEPDC1*
*CRADD*	*DGAT2*
*CREBRF*	*DGKA*
*CREBZF*	*DHFR2*
*CRTAM*	*DHX34*
*CRYGS*	*DLGAP5*
*CSNK1G2*	*DMTF1*
*CTBP1*	*DMTN*
*CTNNA1*	*DNA2*
*CTNNBL1*	*DNAJC15*
*CTSA*	*DNM2*
*CTSW*	*DPH3*
*CWF19L2*	*DPY19L3*
*CXCR6*	*DYNC1I2*
*CXorf65*	*DZIP3*
*CYFIP2*	*E2F5*
*DCAF13*	*EAF2*
*DCP2*	*ECE1*
*DDA1*	*EEF1A1*
*DENND4A*	*EEF1B2*
*DEPDC1*	*EGLN2*
*DGAT2*	*EIF2AK3*
*DGCR2*	*EIF3E*
*DGKA*	*EIF4E*
*DHX34*	*ELAC1*
*DMTF1*	*ELP6*
*DNAAF2*	*EMC2*
*DNAJC15*	*EMG1*
*DNAJC19*	*ENSA*
*DNM2*	*EPB41L4A*
*DPH3*	*EPM2AIP1*
*DPM1*	*ERCC8*
*DPY19L3*	*ERF*
*DYNC1I2*	*ERGIC2*
*DYNC1LI2*	*ERI2*
*DYNLT3*	*ERMARD*
*DZIP3*	*EXOSC10*
*E2F5*	*FAM126A*
*ECE1*	*FAM149B1*
*EEF1A1*	*FAM169A*
*EEF1B2*	*FAM204A*
*EEF1E1*	*FAM3C*
*EFCAB7*	*FANCB*
*EGLN2*	*FARP2*
*EIF2AK3*	*FASTKD1*
*EIF3E*	*FASTKD3*
*EIF4E*	*FAU*
*ELAC1*	*FBXO8*
*ELP6*	*FBXW11*
*EMG1*	*FBXW4*
*ENPP4*	*FCER1A*
*ENSA*	*FCER2*
*EPB41L4A*	*FCGR2B*
*EPM2AIP1*	*FCGR2C*
*EPS15L1*	*FCGRT*
*ERCC8*	*FCRL2*
*ERF*	*FCRLA*
*ERGIC2*	*FFAR2*
*ERI2*	*FGD4*
*ERMARD*	*FGFBP2*
*ESRRA*	*FGGY*
*EVI2A*	*FGR*
*EXOSC10*	*FKBP3*
*EXOSC8*	*FLI1*
*EXOSC9*	*FLII*
*FAM126A*	*FLOT2*
*FAM149B1*	*FNTA*
*FAM169A*	*FOSL2*
*FAM204A*	*FRA10AC1*
*FAM3C*	*FRG1*
*FANCB*	*FRY*
*FANCL*	*FXR1*
*FARP2*	*GCC2*
*FASTKD1*	*GCHFR*
*FAU*	*GCNT2*
*FBXO8*	*GEN1*
*FBXW11*	*GGH*
*FBXW4*	*GIMAP7*
*FCER1A*	*GLOD4*
*FCER2*	*GLS*
*FCGR2B*	*GNL3*
*FCGR2C*	*GPATCH2*
*FCRL2*	*GPD2*
*FCRLA*	*GPM6A*
*FFAR2*	*GPR141*
*FGD4*	*GPR174*
*FGFBP2*	*GPR55*
*FGFR1OP2*	*GPR89A*
*FGGY*	*GPRC5B*
*FGR*	*GPSM3*
*FKBP3*	*GRHL1*
*FLI1*	*GTF2H3*
*FLII*	*GUK1*
*FLOT2*	*GYPA*
*FMNL1*	*GZMA*
*FNTA*	*GZMH*
*FOSL2*	*GZMK*
*FRA10AC1*	*HACD3*
*FRG1*	*HACD4*
*FRY*	*HAT1*
*FXR1*	*HBS1L*
*GATAD2A*	*HCK*
*GBP6*	*HDAC4*
*GCC2*	*HEATR5B*
*GCHFR*	*HENMT1*
*GEMIN2*	*HERPUD2*
*GEN1*	*HIGD1A*
*GFM2*	*HIKESHI*
*GGH*	*HINT1*
*GIMAP7*	*HLA-DOB*
*GLMN*	*HLA-DQA1*
*GLOD4*	*HMGB2*
*GLS*	*HMGN3*
*GNAI1*	*HSF5*
*GNL3*	*HSH2D*
*GPATCH2*	*HSP90AA1*
*GPCPD1*	*HSPA1A*
*GPD2*	*HSPB11*
*GPM6A*	*HSPBAP1*
*GPR141*	*IAH1*
*GPR174*	*IFI44L*
*GPR21*	*IFIT1*
*GPR55*	*IFIT3*
*GPR89A*	*IFNGR1*
*GPRC5B*	*IFT20*
*GPSM3*	*IL15*
*GRK2*	*IL18RAP*
*GSAP*	*IL1RN*
*GTF2H3*	*IL7*
*GTSF1*	*ING3*
*GYPA*	*INTS6L*
*GZMA*	*IQCB1*
*GZMH*	*IQCE*
*GZMK*	*ISG15*
*HABP2*	*ITGA4*
*HACD3*	*ITGB2*
*HACD4*	*ITM2A*
*HACE1*	*ITPR2*
*HAUS1*	*KDM3A*
*HBS1L*	*KDM6B*
*HCK*	*KDM7A*
*HDAC4*	*KIAA0232*
*HEATR5B*	*KIAA0825*
*HERPUD2*	*KIAA0930*
*HIGD1A*	*KIAA1109*
*HINT1*	*KIF15*
*HK1*	*KIF19*
*HLA-DOB*	*KIZ*
*HLA-DQA1*	*KLF13*
*HMGB2*	*KLHDC2*
*HMGN3*	*KLRB1*
*HSF5*	*KLRC3*
*HSH2D*	*KLRC4*
*HSPA14*	*KLRD1*
*HSPB11*	*KLRF1*
*IAH1*	*KLRK1*
*IFIT1*	*KMO*
*IFIT3*	*KRBOX4*
*IFNG*	*KRCC1*
*IFNGR1*	*KRR1*
*IFT20*	*LAMTOR5*
*IL18RAP*	*LANCL1*
*IL1RN*	*LAX1*
*IL5RA*	*LCN2*
*IL7*	*LDAH*
*IMMP2L*	*LDHB*
*ING3*	*LGALS8*
*INTS6L*	*LGALS9*
*IQCB1*	*LILRA6*
*IQCE*	*LLPH*
*IRF2BPL*	*LONRF3*
*ISG15*	*LPXN*
*ITGA4*	*LRFN1*
*ITGAX*	*LRP10*
*ITGB2*	*LSM1*
*ITM2A*	*LSM8*
*ITPR2*	*LSP1*
*KDM3A*	*LTBR*
*KDM6B*	*LTN1*
*KDM7A*	*LTV1*
*KIAA0232*	*LUC7L3*
*KIAA0825*	*LY6E*
*KIAA0930*	*LYN*
*KIAA1109*	*LYRM2*
*KIAA1586*	*LYST*
*KIF18A*	*LYVE1*
*KIF19*	*LZTFL1*
*KIF5C*	*MAGOHB*
*KIZ*	*MAP2K3*
*KLHDC2*	*MAP3K3*
*KLHL9*	*MAP4K3*
*KLRB1*	*MAP7D3*
*KLRC3*	*MAPKAPK2*
*KLRC4*	*MAPKAPK5*
*KLRD1*	*MAPRE2*
*KLRF1*	*MBIP*
*KMO*	*MBNL3*
*KRBOX4*	*MBOAT7*
*KRCC1*	*MCOLN2*
*KRR1*	*MCPH1*
*LAMTOR5*	*MCUB*
*LANCL1*	*MED13*
*LAX1*	*MED14*
*LCN2*	*MELK*
*LDAH*	*MERTK*
*LDHB*	*METTL14*
*LGALS8*	*METTL18*
*LGALS9*	*METTL4*
*LILRA6*	*MIB1*
*LONRF3*	*MICU3*
*LPXN*	*MIDN*
*LRFN1*	*MIER1*
*LRP10*	*MIPEP*
*LSM8*	*MLLT6*
*LTBR*	*MMAA*
*LTN1*	*MMP25*
*LTV1*	*MRPL11*
*LUC7L3*	*MRPL19*
*LYN*	*MRPL22*
*LYRM2*	*MRPL24*
*LYST*	*MRPL35*
*LYVE1*	*MRPL36*
*LZTFL1*	*MRPL40*
*MAP3K3*	*MRPL43*
*MAP4K3*	*MRPL46*
*MAP7D3*	*MRPL47*
*MAPKAPK2*	*MRPL51*
*MAPKAPK5*	*MRPS11*
*MAPRE2*	*MRPS17*
*MBIP*	*MRPS18C*
*MBNL3*	*MS4A3*
*MBOAT7*	*MSN*
*MCAM*	*MTERF1*
*MCOLN2*	*MTERF4*
*MCPH1*	*MTHFD2*
*MCUB*	*MTM1*
*MDH1*	*MVP*
*MED13*	*MX1*
*MED14*	*MYBL1*
*MELK*	*MYC*
*MEMO1*	*MYL12B*
*MERTK*	*MYNN*
*METTL14*	*MYO1F*
*METTL15*	*MYO9A*
*METTL18*	*N4BP2L2*
*METTL2A*	*NAA38*
*METTL4*	*NAA50*
*METTL5*	*NADK*
*MIB1*	*NAP1L4*
*MICU3*	*NBEAL1*
*MIDN*	*NCK1*
*MIER1*	*NCOA3*
*MIPEP*	*NDC80*
*MITD1*	*NDUFA1*
*MLLT6*	*NDUFA4*
*MMAA*	*NDUFA7*
*MMP25*	*NDUFAF4*
*MORF4L2*	*NDUFB1*
*MRPL11*	*NDUFB2*
*MRPL15*	*NDUFB8*
*MRPL19*	*NDUFS3*
*MRPL24*	*NDUFS4*
*MRPL32*	*NEDD4*
*MRPL35*	*NEDD8*
*MRPL39*	*NEIL3*
*MRPL43*	*NEK11*
*MRPL46*	*NFKBIA*
*MRPL50*	*NFU1*
*MRPL58*	*NFXL1*
*MRPL9*	*NFYB*
*MRPS11*	*NIFK*
*MRPS18C*	*NIPAL2*
*MS4A3*	*NIPSNAP3B*
*MS4A6A*	*NLRP1*
*MSN*	*NME8*
*MTCL1*	*NOC3L*
*MTERF1*	*NOL11*
*MTERF4*	*NOP10*
*MTHFD2*	*NOTCH1*
*MTIF3*	*NPM1*
*MVP*	*NPRL3*
*MYBL1*	*NSMCE4A*
*MYC*	*NSUN6*
*MYL12B*	*NUDT7*
*MYNN*	*NUP107*
*MYO1F*	*NUP54*
*MYO1G*	*NUSAP1*
*MYO9A*	*OAS2*
*N4BP2L2*	*OASL*
*NAA38*	*OCIAD2*
*NAA50*	*ODF2L*
*NADK*	*OLA1*
*NAP1L4*	*OLIG1*
*NBEAL1*	*OLIG2*
*NCF1*	*OLR1*
*NCK1*	*OMA1*
*NDUFA7*	*OR2W3*
*NDUFAF4*	*ORC2*
*NDUFB2*	*ORC3*
*NDUFB8*	*OSBPL9*
*NEDD4*	*P2RY10*
*NEK11*	*PANK3*
*NFKBIA*	*PARP15*
*NFXL1*	*PAXBP1*
*NFYB*	*PBX2*
*NIFK*	*PCM1*
*NINJ1*	*PCMTD1*
*NIPSNAP3B*	*PDCD10*
*NIT2*	*PDE4D*
*NLRP1*	*PDE6D*
*NME8*	*PGAM1*
*NOC3L*	*PGBD2*
*NOD1*	*PHF20L1*
*NOL11*	*PHTF2*
*NOP10*	*PIAS1*
*NOTCH1*	*PIGP*
*NPM1*	*PIK3CB*
*NPRL3*	*PKN1*
*NSA2*	*PLAA*
*NSMCE4A*	*PLB1*
*NSUN3*	*PLEKHO2*
*NSUN6*	*PMS1*
*NUCB2*	*PNISR*
*NUDT7*	*PNRC2*
*NUP107*	*POC5*
*NUP54*	*POLA1*
*NUSAP1*	*POLQ*
*OASL*	*POLR2F*
*OCIAD2*	*PPA2*
*ODF2L*	*PPAT*
*ODF3B*	*PPIA*
*OLA1*	*PPIB*
*OLIG1*	*PPIL3*
*OLIG2*	*PPP4C*
*OMA1*	*PPP6R1*
*ORC2*	*PPWD1*
*ORC3*	*PREX1*
*OXER1*	*PRIMPOL*
*OXR1*	*PRKDC*
*OXSM*	*PRPF18*
*P2RY10*	*PRPF40A*
*PANK3*	*PRPS1*
*PARP15*	*PRR3*
*PAXBP1*	*PRRC2C*
*PBDC1*	*PSMA6*
*PBX2*	*PSMC6*
*PCM1*	*PTCD2*
*PCMTD1*	*PTCD3*
*PDCD10*	*PTGDR2*
*PDCL*	*PTGES3*
*PDE4D*	*PTOV1*
*PDE6D*	*PTPN12*
*PDPR*	*PTPN4*
*PDS5A*	*PTRHD1*
*PDZD11*	*PUS3*
*PFKL*	*PXN*
*PGAM1*	*PXYLP1*
*PGBD2*	*RAB12*
*PHF20L1*	*RAB20*
*PHF6*	*RAB28*
*PHTF2*	*RAB35*
*PIAS1*	*RAB3D*
*PIGC*	*RAB3IP*
*PIGP*	*RAB7A*
*PIK3CB*	*RABEP1*
*PIK3CD*	*RABGAP1L*
*PINK1*	*RABIF*
*PKN1*	*RABL3*
*PLAA*	*RAC2*
*PLB1*	*RAD17*
*PLEKHO2*	*RAD51C*
*PLRG1*	*RALGAPA1*
*PMS1*	*RARA*
*PNISR*	*RARS2*
*PNRC2*	*RASA3*
*POC5*	*RASGEF1B*
*POLA1*	*RASSF6*
*POLQ*	*RBM25*
*POLR3F*	*RBM27*
*POU5F2*	*RBM4B*
*PPA2*	*RBM7*
*PPAT*	*RBX1*
*PPIA*	*RETN*
*PPIL3*	*RFESD*
*PPP4C*	*RGS19*
*PPP4R3A*	*RHOBTB3*
*PPP6R1*	*RMDN1*
*PPWD1*	*RNASE2*
*PRELID3B*	*RNASE3*
*PREX1*	*RNF19A*
*PRIMPOL*	*RNF213*
*PRKDC*	*ROMO1*
*PROSER2*	*RPA4*
*PRPF18*	*RPL10A*
*PRPF40A*	*RPL12*
*PRPS1*	*RPL15*
*PRRC2C*	*RPL22*
*PSAP*	*RPL26L1*
*PSMC6*	*RPL29*
*PSMD10*	*RPL30*
*PTCD2*	*RPL34*
*PTCD3*	*RPL35*
*PTGDR2*	*RPL5*
*PTGES3*	*RPRD1A*
*PTPN12*	*RPS15A*
*PTPN22*	*RPS21*
*PTPN4*	*RPS23*
*PTPRC*	*RPS27*
*PXN*	*RPS27A*
*RAB11B*	*RPS29*
*RAB12*	*RPS5*
*RAB20*	*RPS6*
*RAB28*	*RPS6KA1*
*RAB35*	*RRAS2*
*RAB3D*	*RUFY3*
*RAB3IP*	*RWDD2A*
*RAB7A*	*S100A12*
*RABEP1*	*S100Z*
*RABGAP1L*	*SAMD3*
*RABIF*	*SBDS*
*RABL3*	*SBF2*
*RAD17*	*SCCPDH*
*RAD51C*	*SCOC*
*RAD51D*	*SDR39U1*
*RALGAPA1*	*SECC*
*RAN*	*SEL1L*
*RANBP2*	*SENP6*
*RARA*	*SEPSECS*
*RARS2*	*SETDB2*
*RASA2*	*SETX*
*RASA3*	*SF3B1*
*RASSF6*	*SF3B5*
*RBL1*	*SF3B6*
*RBM25*	*SGK1*
*RBM27*	*SH2B3*
*RBM4B*	*SH2D1A*
*RBM7*	*SHISA5*
*RERE*	*SHKBP1*
*RETN*	*SIGLEC6*
*RGS19*	*SIRPA*
*RHOBTB3*	*SKA2*
*RNASE2*	*SKA3*
*RNASE3*	*SLA*
*RNF10*	*SLAMF6*
*RNF135*	*SLC11A2*
*RNF19A*	*SLC25A26*
*RNF213*	*SLC25A29*
*RNFT1*	*SLC25A36*
*ROMO1*	*SLC30A4*
*RPA4*	*SLC38A11*
*RPE*	*SLC5A3*
*RPF1*	*SLCO4C1*
*RPL10A*	*SLIRP*
*RPL12*	*SMAP2*
*RPL15*	*SMC5*
*RPL22*	*SMC6*
*RPL24*	*SMCHD1*
*RPL27A*	*SMDT1*
*RPL29*	*SNRPA1*
*RPL30*	*SNRPD2*
*RPL32*	*SNRPE*
*RPL34*	*SNX13*
*RPL35*	*SNX14*
*RPL36A*	*SNX5*
*RPL37*	*SOAT1*
*RPL5*	*SOX6*
*RPL6*	*SPAG7*
*RPL7*	*SPECC1*
*RPL9*	*SPIDR*
*RPRD1A*	*SPIRE2*
*RPS15A*	*SRP14*
*RPS21*	*SRSF4*
*RPS23*	*SRSF7*
*RPS25*	*SS18*
*RPS27*	*SSR2*
*RPS27A*	*ST3GAL2*
*RPS29*	*ST6GALNAC3*
*RPS3A*	*STAMBP*
*RPS5*	*STAP1*
*RPS6*	*STARD3NL*
*RPS6KA1*	*STAT3*
*RRAGB*	*STAT4*
*RRAS2*	*STK26*
*RSL24D1*	*STRBP*
*RSPH14*	*STXBP2*
*RSRC2*	*SUB1*
*RUFY3*	*SUCLG1*
*RWDD1*	*SUMO1*
*RWDD2A*	*SUPT3H*
*RWDD3*	*SVIP*
*S100A12*	*SYCP2*
*S100Z*	*SYNE2*
*SAMD12*	*TAF12*
*SAMD3*	*TAF2*
*SBDS*	*TANK*
*SBF2*	*TAOK2*
*SCCPDH*	*TARP*
*SCOC*	*TAS2R14*
*SDR39U1*	*TBCA*
*SECC*	*TC2N*
*SECA2*	*TCN1*
*SECTM1*	*TDG*
*SEL1L*	*TECPR2*
*SENP6*	*TEX30*
*SEPSECS*	*TFB2M*
*SETDB2*	*TFEB*
*SF3B1*	*TGFB1*
*SF3B2*	*THAP1*
*SF3B5*	*THOC1*
*SF3B6*	*TIA1*
*SGK1*	*TIAM2*
*SH2B3*	*TIGIT*
*SH2D1A*	*TIMM17A*
*SH3BP1*	*TIMM8B*
*SHISA5*	*TLE3*
*SHKBP1*	*TLN1*
*SIGLEC11*	*TM2D1*
*SIGLEC6*	*TMCC1*
*SIRPA*	*TMCC2*
*SKA2*	*TMEM106C*
*SKA3*	*TMEM116*
*SLA*	*TMEM141*
*SLAMF6*	*TMEM181*
*SLAMF7*	*TMEM258*
*SLC11A2*	*TMEM260*
*SLC25A26*	*TMEM30B*
*SLC25A29*	*TMEM39A*
*SLC25A33*	*TMEM43*
*SLC25A36*	*TMEM60*
*SLC30A4*	*TMEM62*
*SLC35A5*	*TMOD2*
*SLC35D1*	*TMSB15B*
*SLC35E1*	*TMTC3*
*SLC5A3*	*TNFAIP2*
*SLC9B2*	*TNFAIP6*
*SLCO4C1*	*TNFAIP8*
*SLIRP*	*TNFRSF1A*
*SMAP2*	*TOP2B*
*SMC4*	*TPCN1*
*SMC5*	*TPD52L2*
*SMC6*	*TPP2*
*SMCHD1*	*TPR*
*SMDT1*	*TRAM1*
*SMIM20*	*TRIAP1*
*SMIM8*	*TRIM25*
*SNRPA1*	*TRIM61*
*SNRPB*	*TRMO*
*SNRPD1*	*TRMT13*
*SNRPD2*	*TRPS1*
*SNRPE*	*TSC22D3*
*SNX13*	*TTC14*
*SNX14*	*TUBE1*
*SNX24*	*TXN*
*SNX5*	*U2SURP*
*SOAT1*	*UBAP1*
*SOX6*	*UBE2L6*
*SPATA7*	*UBE2M*
*SPECC1*	*UBE2R2*
*SPIDR*	*UBE2T*
*SRFBP1*	*UBE3A*
*SRP14*	*UCHL3*
*SRSF7*	*UFC1*
*SS18*	*UFL1*
*SSB*	*UFSP2*
*ST3GAL2*	*UHRF2*
*ST6GALNAC3*	*UMPS*
*STAMBP*	*UQCRQ*
*STAP1*	*URI1*
*STARD3NL*	*USP16*
*STARD4*	*USP24*
*STAT3*	*USP28*
*STAT4*	*USP45*
*STK26*	*VAMP4*
*STRBP*	*VPS13A*
*STT3A*	*VPS13C*
*STXBP2*	*VPS25*
*SUB1*	*VPS29*
*SUCLG1*	*VPS50*
*SUMO1*	*VSTM1*
*SUPT3H*	*WBP2*
*SVIP*	*WDPCP*
*SYNE2*	*WDR49*
*TAF2*	*WDR61*
*TANK*	*XCL1*
*TAOK2*	*XPO6*
*TARP*	*XRCC4*
*TAS2R14*	*YBX3*
*TBC1D19*	*ZBED5*
*TBCA*	*ZBED6*
*TBL1X*	*ZBED6CL*
*TC2N*	*ZBTB14*
*TCEAL4*	*ZBTB8OS*
*TCEAL8*	*ZC3H8*
*TCEANC2*	*ZDHHC20*
*TCN1*	*ZEB2*
*TECPR2*	*ZFAND1*
*TEX30*	*ZFAND2A*
*TFB2M*	*ZFAND3*
*TFEB*	*ZFC3H1*
*TGFB1*	*ZFP36L1*
*THAP1*	*ZFP37*
*THOC1*	*ZFP69*
*THRAP3*	*ZFP82*
*TIA1*	*ZFYVE16*
*TIGIT*	*ZKSCAN8*
*TIMM17A*	*ZMYM2*
*TLE3*	*ZNF138*
*TLN1*	*ZNF14*
*TM2D1*	*ZNF141*
*TMA16*	*ZNF17*
*TMC5*	*ZNF22*
*TMCC1*	*ZNF227*
*TMCC2*	*ZNF230*
*TMEM116*	*ZNF235*
*TMEM126B*	*ZNF253*
*TMEM181*	*ZNF254*
*TMEM258*	*ZNF260*
*TMEM260*	*ZNF280C*
*TMEM43*	*ZNF280D*
*TMEM62*	*ZNF302*
*TMOD2*	*ZNF32*
*TMSB10*	*ZNF322*
*TMSB15B*	*ZNF420*
*TMTC3*	*ZNF429*
*TNFAIP2*	*ZNF43*
*TNFAIP6*	*ZNF430*
*TNFAIP8*	*ZNF431*
*TNFRSF1A*	*ZNF441*
*TOMM6*	*ZNF467*
*TOP2B*	*ZNF493*
*TOP3B*	*ZNF501*
*TPCN1*	*ZNF506*
*TPD52L2*	*ZNF532*
*TPP2*	*ZNF559*
*TPR*	*ZNF566*
*TRAM1*	*ZNF568*
*TRAPPC6B*	*ZNF569*
*TRIAP1*	*ZNF571*
*TRIM25*	*ZNF585A*
*TRIM61*	*ZNF607*
*TRIM8*	*ZNF626*
*TRMO*	*ZNF638*
*TRMT13*	*ZNF649*
*TRNT1*	*ZNF675*
*TRPS1*	*ZNF678*
*TSC22D3*	*ZNF680*
*TTC14*	*ZNF746*
*TTC30A*	*ZNF782*
*TUBB6*	*ZNF791*
*TUBE1*	*ZNF800*
*TUBGCP4*	*ZNF85*
*TXN*	*ZNF91*
*TYW3*	*ZRANB2*
*U2SURP*	*ZSCAN9*
*UBAP1*	*ZYX*
*UBE2M*	
*UBE2O*	
*UBE2R2*	
*UBE3A*	
*UBR7*	
*UCHL3*	
*UFC1*	
*UFL1*	
*UFSP2*	
*UHRF2*	
*UMPS*	
*UNC50*	
*UNC93B1*	
*UPF1*	
*UQCRQ*	
*URI1*	
*USP16*	
*USP24*	
*USP28*	
*USP31*	
*USP45*	
*VAMP2*	
*VAMP4*	
*VPS13A*	
*VPS13C*	
*VPS25*	
*VPS29*	
*VPS50*	
*VSIG10*	
*VSTM1*	
*WBP2*	
*WDPCP*	
*WDR49*	
*WDR61*	
*WDR7*	
*WEE1*	
*WRN*	
*XCL1*	
*XPO1*	
*XPO6*	
*XRCC4*	
*YEATS4*	
*YOD1*	
*ZBED5*	
*ZBED6*	
*ZBED6CL*	
*ZBTB14*	
*ZBTB8OS*	
*ZC3H8*	
*ZDHHC20*	
*ZEB2*	
*ZFAND1*	
*ZFAND2A*	
*ZFAND3*	
*ZFC3H1*	
*ZFP36L1*	
*ZFP37*	
*ZFP69*	
*ZFP82*	
*ZFYVE16*	
*ZKSCAN8*	
*ZMYM2*	
*ZNF136*	
*ZNF138*	
*ZNF14*	
*ZNF141*	
*ZNF17*	
*ZNF177*	
*ZNF22*	
*ZNF227*	
*ZNF230*	
*ZNF235*	
*ZNF25*	
*ZNF253*	
*ZNF254*	
*ZNF260*	
*ZNF280C*	
*ZNF302*	
*ZNF32*	
*ZNF322*	
*ZNF420*	
*ZNF43*	
*ZNF430*	
*ZNF431*	
*ZNF441*	
*ZNF467*	
*ZNF493*	
*ZNF501*	
*ZNF506*	
*ZNF532*	
*ZNF559*	
*ZNF566*	
*ZNF568*	
*ZNF569*	
*ZNF571*	
*ZNF585A*	
*ZNF607*	
*ZNF626*	
*ZNF638*	
*ZNF649*	
*ZNF658*	
*ZNF675*	
*ZNF678*	
*ZNF680*	
*ZNF746*	
*ZNF782*	
*ZNF784*	
*ZNF791*	
*ZNF800*	
*ZNF85*	
*ZNF91*	
*ZNF93*	
*ZNHIT3*	
*ZRANB2*	
*ZSCAN9*	
*ZYX*	

## Supplementary list 2.

Mitochondrial genes (mitGenes) between comparisons of hypothyroidism, sepsis survivors and sepsis nonsurvivors groups[Table t5]

**Table t5:**

mitGenes in Hypothyroidism and sepsis survivor, n = 95	mitGenes in Hypothyroidism and sepsis nonsurvivor, n = 88
*ABCB7*	*ABCB7*
*ACOT11*	*ACOT11*
*ACP6*	*ACP6*
*ADHFE1*	*ADHFE1*
*ATP23*	*BLOC1S1*
*ATPAF1*	*BOLA3*
*BLOC1S1*	*CASP3*
*BOLA3*	*CBR4*
*CBR4*	*CCDC90B*
*CCDC90B*	*CMC2*
*CMC1*	*CMPK2*
*COA1*	*COA1*
*COQ8A*	*COQ8A*
*COX10*	*COX16*
*COX16*	*COX17*
*COX17*	*COX6A1*
*COX6A1*	*COX6B1*
*COX6B1*	*COX7B*
*COX7B*	*COX7C*
*DNAJC15*	*DBI*
*DNAJC19*	*DNA2*
*FASTKD1*	*DNAJC15*
*GFM2*	*FASTKD1*
*GLOD4*	*FASTKD3*
*GLS*	*GLOD4*
*GPD2*	*GLS*
*HIGD1A*	*GPD2*
*HINT1*	*GUK1*
*IMMP2L*	*HIGD1A*
*KMO*	*HINT1*
*LDHB*	*KMO*
*LYRM2*	*LDHB*
*MCUB*	*LYRM2*
*METTL15*	*MCUB*
*METTL4*	*METTL4*
*METTL5*	*MICU3*
*MICU3*	*MIPEP*
*MIPEP*	*MMAA*
*MMAA*	*MRPL11*
*MRPL11*	*MRPL19*
*MRPL15*	*MRPL22*
*MRPL19*	*MRPL24*
*MRPL24*	*MRPL35*
*MRPL32*	*MRPL36*
*MRPL35*	*MRPL40*
*MRPL39*	*MRPL43*
*MRPL43*	*MRPL46*
*MRPL46*	*MRPL47*
*MRPL50*	*MRPL51*
*MRPL58*	*MRPS11*
*MRPL9*	*MRPS17*
*MRPS11*	*MRPS18C*
*MRPS18C*	*MTERF1*
*MTERF1*	*MTERF4*
*MTERF4*	*MTHFD2*
*MTHFD2*	*NDUFA1*
*MTIF3*	*NDUFA4*
*NDUFA7*	*NDUFA7*
*NDUFAF4*	*NDUFAF4*
*NDUFB2*	*NDUFB1*
*NDUFB8*	*NDUFB2*
*NIPSNAP3B*	*NDUFB8*
*NIT2*	*NDUFS3*
*NSUN3*	*NDUFS4*
*OCIAD2*	*NFU1*
*OMA1*	*NIPSNAP3B*
*OXR1*	*OCIAD2*
*OXSM*	*OMA1*
*PDPR*	*POLQ*
*PINK1*	*PPA2*
*POLQ*	*PRIMPOL*
*PPA2*	*PTCD2*
*PRELID3B*	*PTCD3*
*PRIMPOL*	*RARS2*
*PTCD2*	*RMDN1*
*PTCD3*	*ROMO1*
*RARS2*	*SDR39U1*
*ROMO1*	*SLC25A26*
*SDR39U1*	*SLC25A29*
*SLC25A26*	*SLC25A36*
*SLC25A29*	*SLIRP*
*SLC25A33*	*SMDT1*
*SLC25A36*	*SUCLG1*
*SLIRP*	*TFB2M*
*SMDT1*	*TIMM17A*
*SMIM20*	*TIMM8B*
*SMIM8*	*TRIAP1*
*SUCLG1*	*UQCRQ*
*TFB2M*	
*TIMM17A*	
*TMEM126B*	
*TOMM6*	
*TRIAP1*	
*TRNT1*	
*UQCRQ*	
